# N6-Methyladenosine Modification of CIRCKRT17 Initiated by METTL3 Promotes Osimertinib Resistance of Lung Adenocarcinoma by EIF4A3 to Enhance YAP1 Stability

**DOI:** 10.3390/cancers14225582

**Published:** 2022-11-14

**Authors:** Ying Ji, Qing Zhao, Wei Feng, Yue Peng, Bin Hu, Qirui Chen

**Affiliations:** 1 Department of Thoracic Surgery, Beijing Institute of Respiratory Medicine and Beijing Chao-Yang Hospital, Capital Medical University, Beijing 100020, China; 2 Department of Cardiothoracic Surgery, The Third Xiangya Hospital of Central South University, Changsha 410013, China

**Keywords:** lung adenocarcinoma, osimertinib resistance, circKRT17, m6A modification, YAP1 signaling

## Abstract

**Simple Summary:**

Circular RNA KRT17 (circKRT17) is elevated in osimertinib-resistant lung cancer cells by a circRNA microarray analysis. However, the functional role of circKRT17 in the osimertinib resistance of lung adenocarcinoma (LUAD) remains undetermined. Herein, we found that circKRT17 and METTL3 were elevated in osimertinib-insensitive LUAD tissues and cells. CircKRT17 and METTL3 knockdown increased the sensitivity of LUAD cells to osimertinib. In mechanisms, we demonstrated that METTL3 enhanced the stability of circKRT17 by initiating m6A modification and, thus, promoting YAP1 nuclear localization through the recruitment of EIF4A3. These collective data provide novel insights into therapeutic strategies for osimertinib-resistant LUAD patients. CircKRT17 may act as a biomarker of osimertinib resistance of LUAD and a promising target for osimertinib-resistant LUAD treatment.

**Abstract:**

Background: Circular RNAs (circRNAs) play a key role in regulating the drug resistance of numerous human tumors. However, whether circKRT17 involves in the osimertinib resistance of lung adenocarcinoma (LUAD) remains undetermined. Methods: Relative mRNA/circRNA and protein levels were detected by qRT-PCR and western blotting. Localization of circKRT17 and YAP1 was determined by FISH and immunofluorescence staining. Cell growth and apoptosis were evaluated using colony formation, EdU assays, and flow cytometry. The N6-methyladenosine (m6A) modification was analyzed by MeRIP. The interplay between EIF4A3 and circKRT17 or YAP1 was verified by RNA pull-down or/and RIP assays. Subcutaneous tumor growth was monitored in nude mice, and Ki-67 and TUNEL staining were carried out to evaluate cell proliferation and apoptosis, respectively. Results: CircKRT17 and METTL3 were elevated in osimertinib-insensitive LUAD tissues and cells. Knockdown of circKRT 17 and METTL3 increased the sensitivity of LUAD cells to osimertinib. Knockdown of METTL3 decreased the expression of circKRT17 by inhibiting m6A modification. CircKRT17 promoted the stability and nuclear transportation of YAP1 by recruiting EIF4A3 in LUAD cells. Overexpression of YAP1 abolished the impacts of circKRT17 knockdown on the osimertinib sensitivity of LUAD cells. CircKRT17 knockdown increased the repressive effects of osimertinib on tumor growth in vivo by inhibiting YAP1 signaling. Conclusion: METTL3 initiated the m6A modification of circKRT17, thus promoting osimertinib resistance of LUAD by enhancing YAP1 stability through EIF4A4 recruitment.

## 1. Introduction

Over 80% of all lung cancer cases are non-small cell lung cancer (NSCLC) [1], and lung adenocarcinoma (LUAD) is the most common histological subtype of NSCLC [2]. LUAD patients have frequently been found to carry genetic mutations in the tyrosine kinase domain of the epidermal growth factor receptor (EGFR). Although LUAD patients harboring EGFR mutations have benefited from treatment with EGFR-tyrosine kinase inhibitors (EGFR-TKIs), most patients inevitably develop acquired resistance to EGFR-TKIs, resulting in a poor prognosis [3,4,5]. Osimertinib, as the third generation of oral EGFR-TKI, shows less serious adverse reactions and higher efficacy than the previous two generations of EGFR-TKIs, nevertheless, acquired resistance remains a major challenge for osimertinib treatment [6,7]. Therefore, it is necessary to explore the molecular mechanism of acquired resistance to osimertinib in LUAD to develop a way to alleviate the resistance.

Circular RNAs (circRNAs), a special class of non-coding RNAs, are generated by the mechanisms of “direct back splicing” and “exon skipping” by forming a covalently closed loop [8,9]. During the past decades, numerous dysregulated circRNAs have been identified by bioinformatics analysis in various cell lines and across multiple species [10,11]. These dysregulated circRNAs are proven to affect the expressions of gene products that are involved in tumor biology, such as cell growth, apoptosis, metastasis, drug resistance, and angiogenesis [12,13]. Moreover, the involvement of circRNAs in the tumorigenesis of lung cancer has been well documented [14,15]. CircKRT17 (hsa_circ_0043632), a circRNA that is derived from the Keratin 17 (KRT17) gene, is elevated in osimertinib-resistant lung cancer cells by a circRNA microarray analysis [16]. However, the functional role of circKRT17 in the osimertinib resistance of LUAD remains undetermined.

N6-methyladenosine (m6A) is a common modification in RNA that was originally identified and characterized in the 1970s [17]. Research shows that it is involved in the modulation of the outcome of gene expression by controlling RNA localization, translation, and decay, all of which are regulated by m6A “writers”, “readers”, and “erasers” [18]. RNA methyltransferase methyltransferase-like 3 (METTL3), as an important member of m6A “writers”, has been shown to affect cell proliferation, differentiation, tissue development, and tumorigenesis [19]. Recently, METTL3 was elevated in and associated with the metastasis and cisplatin resistance of lung cancer [20,21]. By using a computational predictor of mammalian m6A site (SRAMP, http://www.cuilab.cn/sramp/), we found m6A sites in the sequence of circKRT17, implying that there may be an interaction between METTL3 and circKRT17. Nevertheless, whether there is an interaction and whether this interaction plays a role in the osimertinib resistance of LUAD remains unclear.

Herein, we demonstrated that circKRT17 was elevated in osimertinib-resistant LUAD. The m6A modification of circKRT17 initiated by METTL3 promoted the nuclear transportation and stability of YAP1 by recruiting EIF4A3, thus promoting osimertinib resistance of LUAD. Our findings proved the role of METTL3/circKRT17/YAP1 in regulating the osimertinib resistance of LUAD and provided novel potential therapeutic targets for osimertinib-resistant LUAD.

## 2. Materials and Methods

### 2.1. Tissue Specimens

Tissues were collected from 24 osimertinib-sensitive LUAD patients and 16 osimertinib-insensitive (resistant) LUAD patients who underwent surgery at the Beijing Institute of Respiratory Medicine and Beijing Chao-Yang Hospital, Capital Medical University. The diagnosis of each patient was verified by a histological examination. Patients with other cancers or who had received chemotherapy or radiotherapy were excluded. Collected tissues were kept at −80 °C until further use. Written informed consent was provided by each participant involved in this study. This research was approved by the Ethics Committee of Beijing Chao-Yang Hospital of Capital Medical University.

### 2.2. Cell Culture

H1975 and PC-9 were obtained from the Chinese Academy of Sciences Collection Committee cell bank (Shanghai, China) and maintained under 37 °C in RPMI-1640 containing 4 mM L-glutamine (Invitrogen, Carlsbad, CA, USA), fetal bovine serum (10%, Invitrogen Carlsbad, CA, USA), and penicillin/streptomycin (1%, Invitrogen).

### 2.3. Generation of Osimertinib-Resistant LUAD Cell Lines

H1975/OR and PC-9/OR were generated from their parental cell lines by gradually increasing the osimertinib concentration. In brief, parental cells (1 × 10^6^) were plated onto a cell culture dish (10 cm^2^) and treated with osimertinib, starting from 500 nM. Osimertinib concentration was increased by 500 nM every 15 days until the final concentration of 2 µM was reached. Osimertinib-treated cells were further plated on 96-well plates using limiting dilution and a single clone was selected and maintained in culture medium containing 2 µM of osimertinib.

### 2.4. Plasmid Construction and Transfection

Specific short hairpin RNAs (shRNAs) were generated to knock down the expression of the target gene. Two specific shRNAs targeted circKRT17 and METTL3, and were inserted into the pGLVH1 vector (GenePharm, Shanghai, China) to generate shcircKRT17#1, shcircKRT17#2, shMETTL3#1, and shMETTL3#2. The indicated lentiviral vector was transfected into HEK293T cells together with package plasmid by Lipofectamine 3000 (Invitrogen, Carlsbad, CA, USA), and puromycin (2 μg/mL) was applied to screen target cells. The full-length circKRT17 and YAP1 sequences were inserted into the pcDNA3.1 vector (GenePharm, Shanghai, China) to overexpress circKRT17 and YAP1, respectively.

### 2.5. Fluorescence In Situ Hybridization (FISH)

The FISH assay was performed to detect the subcellular localization of circKRT17 in H1975/OR cells. An in situ hybridization kit (GenePharma, Shanghai, China) was used in this experiment. The specific Cy3-labeled circKRT17 probe was provided by Roche (Indianapolis, IN, USA). In brief, after fixation in 4% formaldehyde and washing in PBS, LUAD cells were subjected to hybridization with the Cy3-labeled circKRT17 probe overnight. Signals were imaged using a confocal microscope (Olympus, Tokyo, Japan).

### 2.6. Treatment with Actinomycin D and RNase R

Since circRNA does not have 5 and 3 ends, it is resistant to exonuclease-mediated degradation and, thus, more stable than linear RNA. To confirm the circular structure of circKRT17, Actinomycin D and RNase R were used to treat circKRT17 and linear KRT17 mRNA. After treatment with Actinomycin D (5 μg/mL, Sigma, St Louis, MO, USA), the RNA levels of circKRT17 and linear KRT17 mRNA were examined by qRT-PCR, as described above. For RNase R treatment, total RNAs were divided into two parts, one for RNase R (Epicenter Technologies, Madison, WI, USA) incubation and the other for non-incubation (mock). The RNA expressions of circKRT17 and linear KRT17 mRNA were examined by qRT-PCR, as described above.

### 2.7. Calculation of IC_50_ Value Using MTT Assay

In order to calculate the IC50 values of H1975, PC-9, H1975/OR, PC-9/OR cells to osimertinib, an MTT assay was performed to detect the cell viability after the treatment with different concentrations of osimertinib. H1975, PC-9, H1975/OR, and PC-9/OR cells (1 × 10^5^) were plated on 96-well plates, and osimertinib (0.001 μM, 0.01 μM, 0.1 μM, 1 μM, and 10 μM) was added. After 48 h of incubation with osimertinib, methyl thiazolyl tetrazolium (MTT) reagent (0.5 mg/mL, Sigma) was added and incubated for 4 h. Finally, absorbance at 490 nm was measured in a microplate reader (Bio-Rad, Hercules, CA, USA). IC_50_ represents the concentration of osimertinib that is required for half repression of cell growth.

### 2.8. Colony Formation Assay

A colony formation assay was conducted to assess the cell proliferation of the treated cells. Treated LUAD cells were collected and seeded on six-well plates and cultured in culture medium supplied with 10% fetal bovine serum for 14 days. Proliferating colonies were fixed in methanol and then stained with 1% crystal violet (Beyotime, Shanghai, China). The colony number was counted under a microscope (Olympus, Tokyo, Japan).

### 2.9. EdU Immunofluorescence Staining

EdU immunofluorescence staining was performed to further evaluate cell proliferation. An EdU kit (Beyotime) was employed in this experiment. The treated cells (2 × 10^4^) were seeded and cultured on 96-well plates for 24 h. Afterward, 50 μM EdU was added and incubated for 2 h, followed by the incubation with 4% paraformaldehyde and 0.5% Triton X-100. Finally, cells were stained with 1 × Apollo^®^ reaction cocktail for 30 min. Signals were detected under a confocal microscope (Olympus, Tokyo, Japan).

### 2.10. Flow Cytometry Analysis of Cell Apoptosis

The apoptosis rate of the treated cell was detected by flow cytometry analysis. An Annexin-V/FITC and propidium iodide (PI) detection kit (BD Biosciences, San Jose, CA, USA) was adopted in this study to assess the apoptosis percentage of treated LUAD cells following the protocol of the manufacturer. The apoptosis rate was analyzed by flow cytometry (BD Bioscience, San Jose, CA, USA).

### 2.11. Immunofluorescence Analysis

Immunofluorescence analysis was performed to detect the expression of YAP1 in LUAD cells. After fixation with 4% paraformaldehyde for 24 h, treated LUAD cells were washed in PBS and incubated with 5% BSA for 1 h, followed by the incubation with anti-YAP1 antibody (Rabbit, ab52771, 1:500, Abcam) overnight at 4 °C. After three washes, cells were stained with donkey–anti-rabbit 647 (1:200, ab150075, Abcam). Nuclear was stained with DAPI. Fluorescence images were acquired by a confocal microscope (Olympus).

### 2.12. RNA Immunoprecipitation (RIP)

A RIP assay was performed to test the interaction among circKRT17, YAP1, and EIF4A3 in LUAD cells. A Magna RIP kit (EMD Millipore, Billerica, MA, USA), anti-EIF4A3, and IgG (used as control) were adopted in this experiment. Co-precipitated RNAs were isolated and examined by qRT-PCR. Total RNAs (input control) were also detected.

### 2.13. RNA Pull-Down

An RNA pull-down assay was utilized to determine the interaction between circKRT17 and EIF4A3 using biotin-labeled circKRT17. Biotin-labeled circKRT17 was designed and provided by GenePharm (Shanghai, China). A specific circKRT17 probe was mixed and incubated with cell lysates of the indicated LUAD cells overnight. Next, streptavidin-conjugated magnetic beads (Invitrogen, Carlsbad, CA, USA) were added and incubated for another 2 h. Beads were then rinsed and interested protein levels were detected via western blotting.

### 2.14. MeRIP

A MeRIP assay was adopted to detect the m6A methylation level of circKRT17 in LUAD cells. Briefly, isolated mRNAs were chemically shredded into ~100 nucleotide fragments by Ambion reagent (Thermo Fisher Scientific, Waltham, MA, USA). Next, these fragments were denatured for 5 min at 65 ℃ and incubated with Magnetic Beads (A + G, 2923270, Millipore, Billerica, MA, USA) conjugated to anti-m6A antibody (ab208577, Abcam). mRNA was allowed to incubate with m6A-bound beads for 4 h in IPP buffer (15 mM NaCl, 0.1% NP-40, 10 mM Tris-HCl). RLT buffer was employed to elute RNA from the beads and then purified using Qiagen RNeasy kit (Qiagen, Shanghai, China). Relative abundances of the indicated RNA were measured by qRT-PCR.

### 2.15. Quantitative Real-Time PCR (qRT-PCR)

Relative expressions of circKRT17, METTL3, and YAP1 mRNA in LUAD cells were tested by qRT-PCR. Total RNA isolation was performed using TRIzol (Invitrogen). After determining the quality and concentration of the extracted RNA, it was reversely transcribed into cDNA by a PrimeScript™ RT Kit (Takara, Tokyo, Japan). The PCR process was carried out on an ABI 7500 Real-Time System (Applied Biosystems, Foster City, CA, USA) using a YBR Green kit (Roche). Primers were synthesized by RiboBio (Guangzhou, Guangdong, China). The transcriptional levels of targeted genes were analyzed by the 2^−ΔΔCt^ method. The following primers were used in this study: GAPDH: (F: 5-CCAGGTGGTCTCCTCTGA-3 and R: 5-GCTGTAGCCAAATCGTTGT-3); circKRT17: (F: 5-TCCCTTCCCCATGCTTCCTT-3 and R: 5-GCGGGAGGAGATGACCTTG-3); METTL3: (F: 5-GAGTGCATGAAAGCCAGTGA-3 and R: 5-CTGGAATCACCTCCGACACT-3); YAP1: (F: 5-CACAGCATGTTCGAGCTCAT-3 and R: 5-CTCAGCGTCCTGGGTTACAT-3).

### 2.16. Western Blot Analysis

Protein expression levels in treated LUAD cells were measured by a western blot analysis. Proteins of tissues and cells were prepared by a RIPA lysis buffer (Thermo Fisher, Waltham, MA, USA). After the determination of the quality and concentration, proteins were loaded into 10% SDS-PAGE for isolation, followed by the transfer to nitrocellulose membranes. The membranes were subjected to incubation with 5% low-fat milk for 2 h for blocking the non-specific binding. We rinsed the membranes with PBS (three times) and then incubated them with primary antibodies against METTL3 (ab195352, 1:1000, Abcam, Cambridge, MA, USA), YAP1 (ab52771, 1:5000, Abcam), p-TAZ (sc-17610, 1:2000, Santa Cruz Biotechnology, SCB, Santa Cruz, CA, USA), TAZ (sc-518206, 1:1000, SCB), P-gp (ab262880, 1:5000, Abcam), MRP-1 (ab260038, 1:1000, Abcam), c-Myc (ab32072, 1:000, Abcam), Cyclin D1 (ab16663, 1:200, Abcam), Bax (ab32503, 1:5000, Abcam), Bcl2 (ab182858, 1:2000, Abcam), and GAPDH (ab8245, 1:10000, Abcam) for 24 h. Afterward, the membranes were incubated with indicated secondary antibodies (Abcam) for 2 h; the bands were detected by an enhanced chemiluminescence reagent (EMD Millipore, Billerica, MA, USA).

### 2.17. Xenograft Tumor Growth Assay

The xenograft tumor growth assay was performed to test the effects of circKRT17 knockdown on LUAD progression in vivo. Nude mice (6-weeks old, male, SJA Laboratory Animal Co., Ltd., Changsha, Hunan, China) were used in this study. Animal procedures were approved by the Ethics Committee of Beijing Chao-Yang Hospital of Capital Medical University. H1975/OR and PC-9/OR cells (2 × 10^6^) stably transfected with shNC or shcircKRT17 were subcutaneously delivered into the left flanks of the mice. When the tumor volume reached 50 mm^3^, mice were treated with osimertinib (25 mg/kg/d) or noting by oral gavage for 14 days. Tumors were examined every 3 days by calipers, and the volume was calculated by the formula: (length × width^2^)/2. In the end, animals were sacrificed and tumors were collected for further study.

### 2.18. Immunohistochemistry and TUNEL Staining

An immunohistochemistry analysis of Ki-67 was performed to assess the cell proliferation of LUAD cells; TUNEL staining was adopted to evaluate the cell apoptosis of LUAD cells. Isolated tumors were fixed in 4% paraformaldehyde overnight. Afterward, tumors were rinsed with pre-cold PBS and embedded in paraffin, and cut into 4-μm sections using a microtome. After antigen retrieval with sodium citrate buffer, tumor sections were incubated overnight with an anti-Ki-67 antibody (Cell Signaling Technology, Beverly, MA, USA). Tissues were also stained with DAPI (1:10,000, 5 min) to stain nuclear. Signals were detected using a confocal microscope (Olympus, Tokyo, Japan). TUNEL staining of tumor sections was performed using an in situ cell death detection fluorescein kit (Roche) following the manufacturer’s instructions. Signals were detected using a microscope (Olympus, Tokyo, Japan).

### 2.19. Statistical Analysis

All analyses were done in GraphPad Prism 7 (GraphPad Software Inc.). Data are presented as Mean ± SD. The unpaired two-tailed Student’s t test or/and one-way ANOVA with Turkey post hoc test were performed to analyze the differences between two or multiple groups. The statistical significance was defined as a *p* value of less than 0.05.

## 3. Results

### 3.1. CircKRT17 Was Elevated in Osimertinib-Resistant LUAD

**Figure 1:**
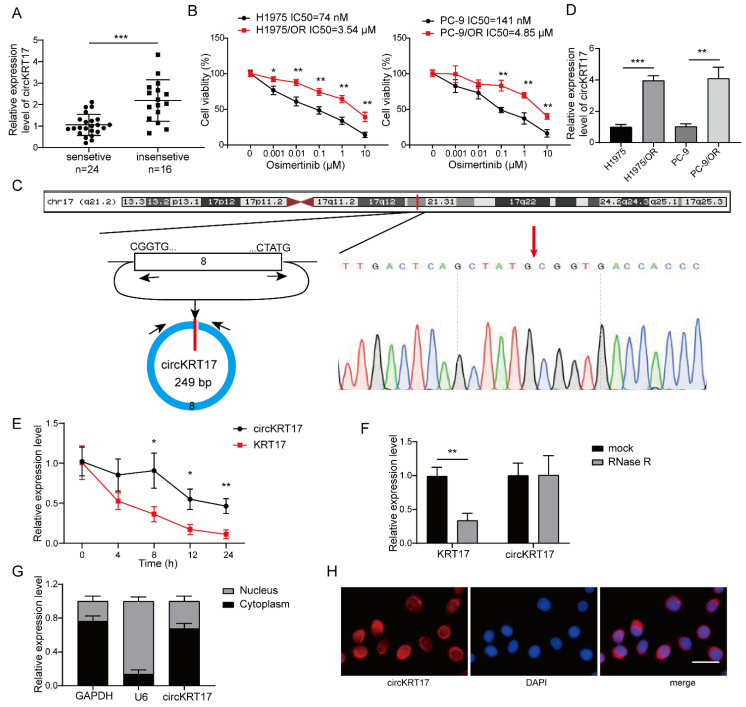
CircKRT17 was elevated in osimertinib-resistant LUAD. (**A**) Relative circKRT17 level in the tissues from osimertinib-sensitive and -insensitive LUAD patients. (**B**) Half-maximal inhibitory concentration (IC_50_) value of osimertinib in H1975 and PC-9 and H1975/OR and PC-9/OR. (**C**) Relative circKRT17 level in H1975 and PC-9 and H1975/OR and PC-9/OR. (**D**) Schematic diagram of the gene location and formation of circKRT17. Relative levels of circKRT17 and KRT17 mRNA in H1975/OR cells after treatment with (**E**) Actinomycin D or (**F**) RNase R. (**G**) The abundance of circKRT17 in either the cytoplasm or nuclear of H1975/OR cells. U6 and GAPDH were employed as controls. (**H**) RNA FISH was performed to identify the subcellular localization of circKRT17 in H1975/OR cells. * *p* < 0.05, ** *p* < 0.01 and *** *p* < 0.001.

To explore whether circKRT17 has a role in LUAD resistance to osimertinib, we first tested the level of circKRT17 in lung tissues collected from osimertinib-sensitive (*n* = 24) and -insensitive (*n* = 16) LUAD patients. Compared to the osimertinib-sensitive group, circKRT17 was elevated in the osimertinib-insensitive group (Figure 1A). The osimertinib-resistant LUAD cell lines (H1975/OR and PC-9/OR) were generated from their parental cell lines by elevating the osimertinib concentration gradually. Half-maximal inhibitory concentration (IC_50_) values of osimertinib for H1975/OR and PC-9/OR cells were 3.54 µM and 4.85 µM, respectively, while the IC50 value for H1975 and PC-9 was only 74 nM and 141 nM, respectively (Figure 1B). We also found a higher expression of circKRT17 in H1975/OR and PC-9/OR cells than in H1975 and PC-9 cells (Figure 1C). CircKRT17 (hsa_circ_0043632) was transcribed from the exon 8 of the KRT17 gene by back-splicing with a length of 249 bp, and the junction site was further validated by Sanger sequencing (Figure 1D). Actinomycin D exposure experiment showed that the half-life of circKRT17 exceeded 24 h while the linear KRT17 mRNA was less than 4 h in the presence of actinomycin D (Figure 1E), indicating that circKRT17 was more stable than linear KRT17 mRNA. Moreover, we found that circKRT17, but not linear KRT17 mRNA, had resistance to RNase R digestion, suggesting a typical loop structure of circKRT17 (Figure 1F). In addition, the subcellular localization of circKRT17 was detected using nucleoplasmic separation followed by qRT-PCR and FISH experiments. Results indicated that circKRT17 was preferentially localized in the cytoplasm (Figure 1G,H). These results indicated that circKRT17 was a stable circular transcript that elevated in osimertinib-resistant LUAD.

### 3.2. Knockdown of CIRCKRT17 Increased the Sensitivity of Osimertinib-Resistant LUAD Cells to Osimertinib

**Figure 2:**
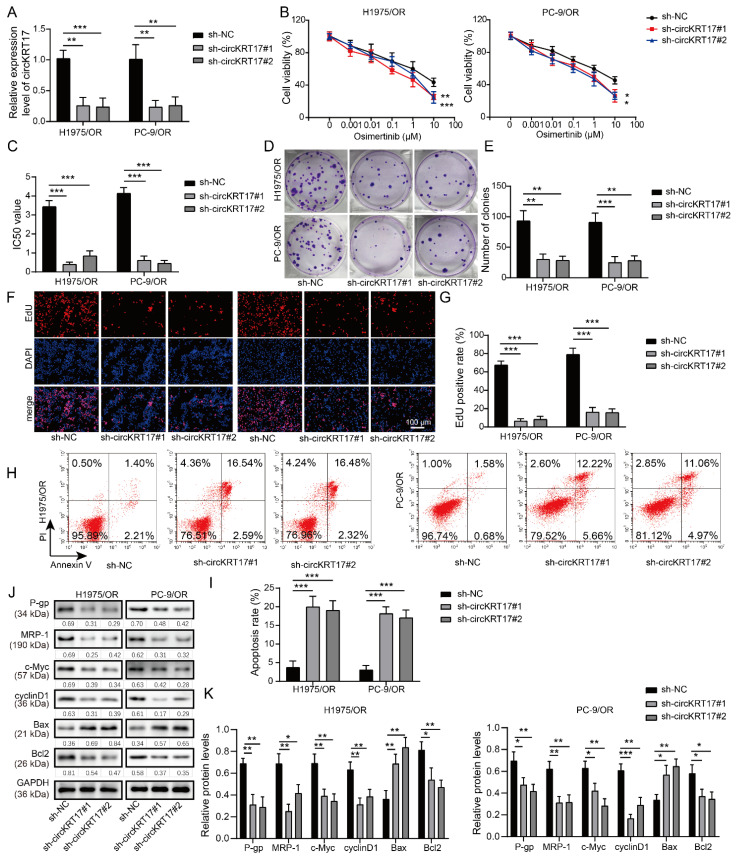
Knockdown of circKRT17 increased the sensitivity of osimertinib-resistant LUAD cells to osimertinib. (**A**) Two shRNAs target circKRT17 (shcircKRT17#1 and shcircKRT17#2) were designed to knock down the expression of circKRT. (**B**,**C**) Osimertinib IC50 value of H1975/OR and PC-9/OR cells transfected with shNC, shcircKRT17#1 and shcircKRT17#2. (**D**,**E**) Colony formation assay in H1975/OR and PC-9/OR cells transfected with shNC, shcircKRT17#1 and shcircKRT17#2. (**F**,**G**) EdU immunofluorescence staining was conducted to evaluate cell proliferation of H1975/OR and PC-9/OR cells from different groups. (**H**,**I**) Effects of circKRT17 knockdown on H1975/OR and PC-9/OR cell apoptosis assessed via flow cytometry. (**J**,**K**) Western blot analysis of P-gp, MRP-1, c-Myc, CyclinD1 Bax, and Bcl2 in H1975/OR and PC-9/OR cells transfected with shNC, shcircKRT17#1 and shcircKRT17#2. * *p* < 0.05, ** *p* < 0.01 and *** *p* < 0.001. File S1: Original western blots.

To understand the functions of circKRT17 in osimertinib resistance, two shRNAs target circKRT17 (shcircKRT17#1 and shcircKRT17#2) were designed to knock down the expression of circKRT17. Both shcircKRT17#1 and shcircKRT17#2 could significantly decrease the expression of circKRT17 in H1975/OR and PC-9/OR cells compared to the shNC group (Figure 2A). The MTT assay suggested that transfection with shcircKRT17#1 and shcircKRT17#2 reduced the IC_50_ value of osimertinib for H1975/OR and PC-9/OR cells (Figure 2B,C). The colony numbers in shcircKRT17#1 and shcircKRT17#2 transfected groups were dramatically reduced compared to that in the shNC group (Figure 2D,E). Consistently, by using EdU immunofluorescence staining, less proliferative cells were observed in H1975/OR and PC-9/OR cells with circKRT17 knockdown compared with the shNC group (Figure 2F,G). Effects of circKRT17 silencing on apoptosis were evaluated by flow cytometry. Results indicated that circKRT17 knockdown promoted the apoptosis of H1975/OR and PC-9/OR cells (Figure 2H,I). Additionally, we detected the levels of multidrug resistance and survival-associated proteins, namely P-gp, MRP-1, c-Myc, Cyclin D1, Bax, and Bcl2. Knockdown of circKRT17 could reduce the expression of multidrug resistance-related protein P-gp and MRP-1 (Figure 2J,K). Moreover, the levels of pro-survival proteins c-Myc, Cyclin D1, and Bcl2 were reduced while that of pro-apoptotic protein Bax was increased in circKRT17 silenced H1975/OR and PC-9/OR cells (Figure 2J,K). These findings suggested that the knockdown of circKRT17 increased the sensitivity of osimertinib-resistant LUAD cells to osimertinib.

### 3.3. Elevated METTL3 in LUAD Regulated CIRCKRT17 Expression by m6A Modification

**Figure 3:**
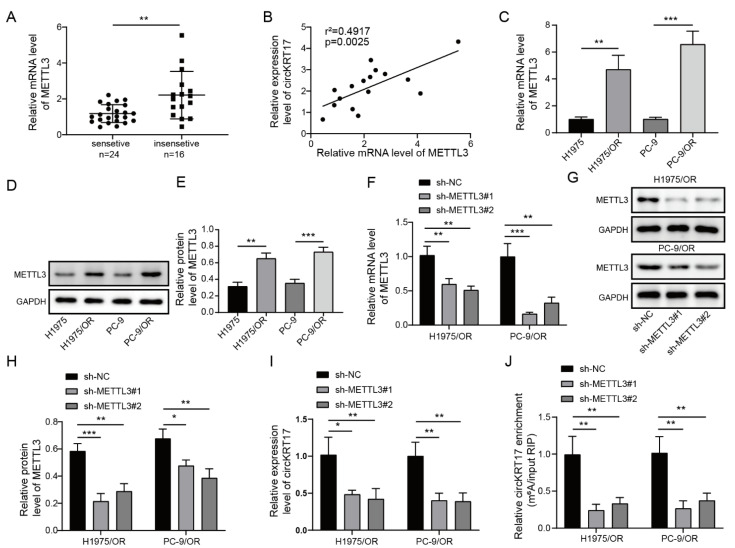
Elevated METTL3 in LUAD regulated circKRT17 expression by m6A modification. (**A**) Relative METTL3 mRNA expression in the tissues from osimertinib-sensitive and -insensitive LUAD patients. (**B**) Correlation analysis between the levels of circKRT17 and METTL3 mRNA. Relative (**C**) mRNA and (**D**,**E**) protein expression of METTL3 in H1975 and PC-9 and H1975/OR and PC-9/OR. Two shRNAs target METTL3 (shMETTL3#1 and shMETTL3#2) were designed to knock down the expression of METTL3, knockdown efficiency was tested using (**F**) qRT-PCR and (**G**,**H**) western blotting. (**I**) Relative circKRT17 expression in H1975/OR and PC-9/OR cells transfected with shNC, shMETTL3#1 and shMETTL3#2. (**J**) The m6A methylation level of circKRT17 in H1975/OR and PC-9/OR cells transfected with shNC, shMETTL3#1, and shMETTL3#2. * *p* < 0.05, ** *p* < 0.01 and *** *p* < 0.001. File S1: Original western blots.

CircRNAs are regulated by METTL3 through m6A modification in various human diseases. Thus, we examined the expression of METTL3 in lung tissues collected from osimertinib-sensitive (*n* = 24) and -insensitive (*n* = 16) LUAD patients. METTL, compared to the osimertinib-sensitive group, was elevated in the osimertinib-insensitive group (Figure 3A). We also detected a positive correlation between the levels of circKRT17 and METTL3 mRNA (Figure 3B). Consistently, METTL3 was found to be elevated in H1975/OR and PC-9/OR cells compared to H195 and PC-9 cells in both mRNA (Figure 3C) and protein levels (Figure 3D,E). To determine whether METTL3 could regulate circKRT17 expression, two shRNAs targeting METTL3 (shMETTL3#1 and shMETTL3#2) were designed to knock down the expression of METTL3 in H1975/OR and PC-9/OR cells. Transfection with shMETTL3#1 or shMETTL3#2 dramatically decreased the mRNA (Figure 3F) and protein (Figure 3G,H) expression of METTL3 compared to shNC transfection, suggesting that METTL3 was successfully silenced by shMETTL3#1 and shMETTL3#2. Knockdown of METTL3 in H1975/OR and PC-9/OR cells significantly decreased the expression of circKRT17 (Figure 3I). Moreover, by using MeRIP-qPCR, we found a significant decrease in circKRT17 m6A methylation in METTL3 knockdown H1975/OR and PC-9/OR cells (Figure 3J). These findings suggested that elevated METTL3 in osimertinib-resistant LUAD regulated the expression of circKRT17 by m6A modification.

### 3.4. Knockdown of METTL3 Increased the Sensitivity of Osimertinib-Resistant LUAD Cells to Osimertinib by Inhibiting CIRCKRT17

**Figure 4:**
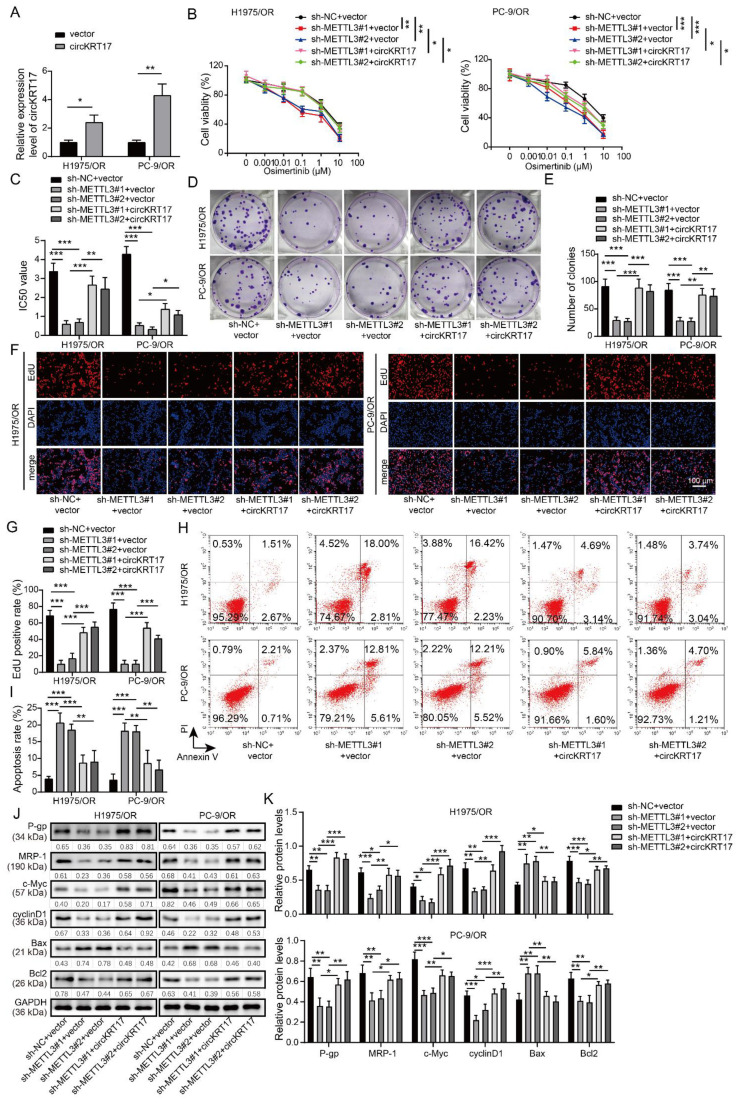
Knockdown of METTL3 increased the sensitivity of osimertinib-resistant LUAD cells to osimertinib by inhibiting circKRT17. (**A**) CircKRT17 overexpression plasmid was designed to overexpress circKRT17 in osimertinib-resistant LUAD cells, and overexpression efficiency was detected by Qrt-PCR. (**B**,**C**) Osimertinib IC50 value of H1975/OR and PC-9/OR cells transfected with shMETTL3#1 or shMETTL3#2 and circKRT17 overexpression plasmid. (**D**,**E**) Colony formation assay and (**F**,**G**) EdU immunofluorescence staining were performed to detect cell proliferation after treatment with shMETTL3#1 or shMETTL3#2 and circKRT17 overexpression plasmid. (**H**,**I**) After co-transfection with shMETTL3#1 or shMETTL3#2 and circKRT17 overexpression plasmid, H1975/OR and PC-9/OR cells were subjected to apoptosis analysis using flow cytometry. (**J**,**K**) Western blot analysis of P-gp, MRP-1, c-Myc, CyclinD1 Bax, and Bcl2 in H1975/OR and PC-9/OR cells transfected with shMETTL3#1 or shMETTL3#2 and circKRT17. * *p* < 0.05, ** *p* < 0.01 and *** *p* < 0.001. File S1: Original western blots.

Next, rescue assays were designed to determine the role of the METTL3/circKRT17 axis in the resistance of H1975/OR and PC-9/OR cells to osimertinib. A circKRT17 overexpression plasmid was used to overexpress circKRT17, and qRT-PCR results indicated significant upregulation of circKRT17 in both H1975/OR and PC-9/OR cells transfected with circKRT17 overexpression plasmid compared to the vector group (Figure 4A). Knockdown of METTL3 by shMETTL3#1 and shMETTL3# could decrease the IC_50_ value of osimertinib for H1975/OR and PC-9/OR cells, while overexpression of circKRT17 could reverse the above effects in METTL3-silenced cells (Figure 4B,C). By using the colony formation assay (Figure 4D,E) and EdU immunofluorescence staining (Figure 4F,G), we revealed an inhibition of cell proliferation in METTL3-silenced cells, while circKRT17 overexpression abolished the inhibitive effects of METTL3 knockdown on cell proliferation. The promotive effects of METTL3 knockdown on cell apoptosis were also abrogated by circKRT17 overexpression (Figure 4H,I). Knockdown of METTL3 decreased the expression of multidrug resistance-related proteins (P-gp and MRP-1) and pro-survival proteins (c-Myc, Cyclin D1, and Bcl2) and increased the expression of pro-apoptotic protein Bax, moreover, the alterations of these proteins induced by METTL3 knockdown were reversed by circKRT17 overexpression (Figure 4J,K). These results suggested that the knockdown of METTL3 increased the sensitivity of H1975/OR and PC-9/OR cells to osimertinib by inhibiting circKRT17.

### 3.5. CIRCKRT17 Promoted the Expression and Nuclear Transportation of YAP1

**Figure 5:**
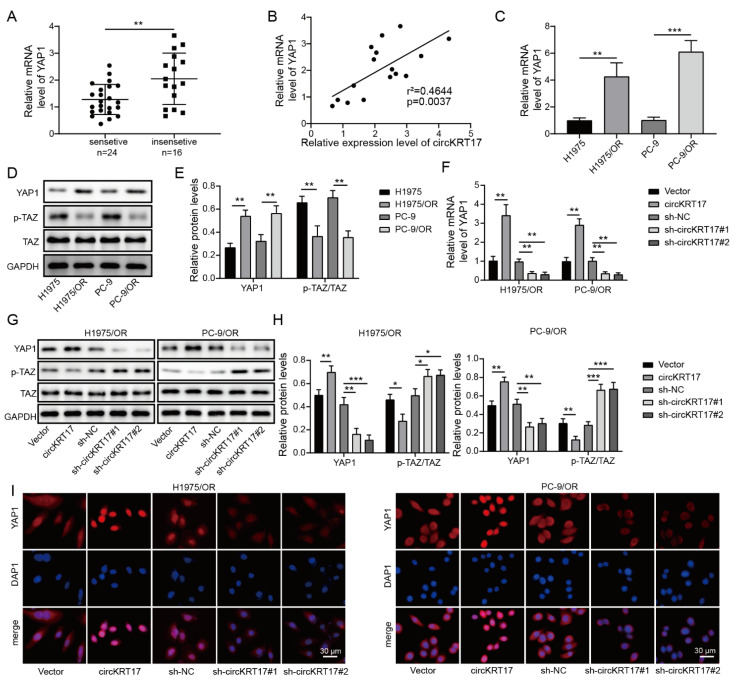
CircKRT17 promoted the expression and nuclear transportation of YAP1. (**A**) Relative YAP1 mRNA expression in the tissues from osimertinib-sensitive and –insensitive LUAD patients. (**B**) Correlation analysis between the levels of YAP1 mRNA and circKRT17 in LUAD tissue. Relative (**C**) mRNA and (**D**,**E**) protein expression of YAP1 in H1975 and PC-9 and H1975/OR and PC-9/OR. (**F**) Relative YAP1 mRNA level in H1975/OR and PC-9/OR cells transfected with circKRT17, shcircKRT17#1, or shcircKRT17#2. (**G**,**H**) Western blot analysis of YAP1, p-TAZ, and TAZ protein levels in H1975/OR and PC-9/OR cells transfected with circKRT17, shcircKRT17#1 or shcircKRT17#2. (**I**) Immunofluorescence analysis of YAP1 in H1975/OR and PC-9/OR cells transfected with circKRT17, shcircKRT17#1, or shcircKRT17#2. * *p* < 0.05, ** *p* < 0.01 and *** *p* < 0.001. File S1: Original western blots.

Yes-associated protein 1 (YAP1) has been reported to be elevated in various solid tumors and plays a critical role in tumorigenesis [22,23,24]. Here, we also found a significant elevation of YAP1 mRNA in the tissues of osimertinib-insensitive patients (Figure 5A). We also found a positive correlation between YAP1 mRNA and circKRT17 levels (Figure 5B). Consistently, YAP1 was revealed to be elevated in H1975/OR and PC-9/OR cells in mRNA (Figure 5C) and protein levels (Figure 5D,E). Overexpression of circKRT17 by overexpression plasmid could dramatically increase the mRNA level of YAP1 while knockdown of circKRT17 by shcircKRT17#1 and shcircKRT17#2 could sharply decrease the mRNA level of YAP1 (Figure 5F). Results from western blotting suggested that overexpression of circKRT17 increased YAP1 protein level and decreased p-TAZ protein level, while knockdown of circKRT17 had the opposite effect (Figure 5G,H). Immunofluorescence analysis showed that more YAP1 was present in the nucleus when circKRT17 was overexpressed, and less YAP1 was present in the nucleus when circKRT17 was silenced (Figure 5I). These findings implied that circKRT17 activated the YAP1/TAZ signaling pathway by accelerating YAP1 nuclear transport.

### 3.6. CIRCKRT17 Enhanced the Stability of YAP1 mRNA by Recruiting EIF4A3

**Figure 6:**
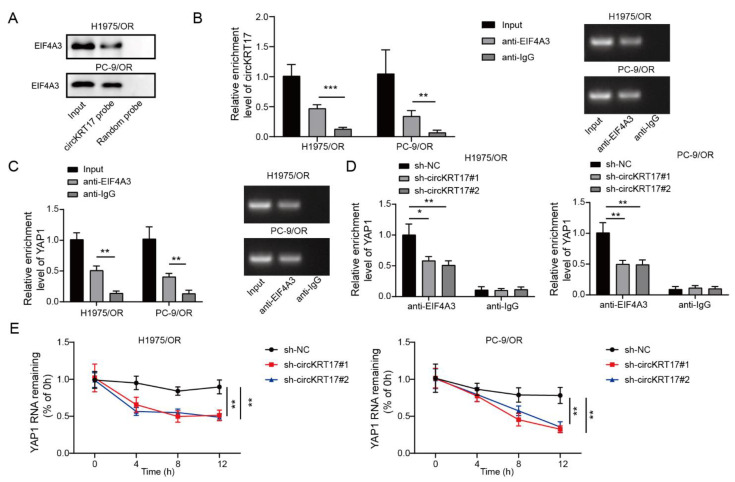
CircKRT17 enhanced the stability of YAP1 mRNA by recruiting EIF4A3. (**A**) Verification of the interplay of circKRT17 and EIF4A3 using a circKRT17 probe. (**B**,**C**) Interplay between EIF4A3 and circKRT17 or YAP1 was verified by the RIP assay in H1975/OR and PC-9/OR cells. (**D**) Impacts of circKRT17 silencing on the interaction of EIF4A3 and YAP1 were evaluated via RIP assay. (**E**) Relative YAP1 mRNA level in H1975/OR and PC-9/OR cells transfected with shNC, shcircKRT17#1 or shcircKRT17#2 in the presence of Actinomycin D. * *p* < 0.05, ** *p* < 0.01 and *** *p* < 0.001. File S1: Original western blots.

By CircInteractome (https://circinteractome.nia.nih.gov/, accessed on 13 March 2020) and Starbase (https://starbase.sysu.edu.cn/index.php, accessed on 13 March 2020), we found EIF4A3 protein binding sites in both circKRT17 and YAP1 mRNA. Given the important role of EIF4A3 in regulating mRNA splicing, translation, and degradation, and the increasing evidence of circRNAs interacting with EIF4A3, we hypothesized that circKRT17 might regulate YAP1 mRNA by interacting with EIF4A3. Thus, we first tested the interplay between circKRT17 and EIF4A3 using a specific circKRT17 probe. Results indicated that EIF4A3 could be pulled down by the circKRT17 probe (Figure 6A). Consistently, in the RIP assay, circKRT17 and YAP1 were found to be enriched by anti-EIF4A3 antibody but not by anti-IgG (Figure 6B,C). Moreover, we found that circKRT17 knockdown in H1975/OR and PC-9/OR cells could inhibit the enrichment of YAP1 by anti-EIF4A3 (Figure 6D). Actinomycin D incubation assay showed that YAP mRNA was less stable in circKRT17 silenced cells compared to that in shNC-treated cells (Figure 6E). These findings proved that the knockdown of circKRT17 inhibited the stability of YAP1 mRNA by recruiting EIF4A3.

### 3.7. YAP1 Overexpression Reversed the Promotive Effects of CIRCKRT17 Knockdown on the Sensitivity of Osimertinib-Resistant LUAD Cells to Osimertinib

**Figure 7:**
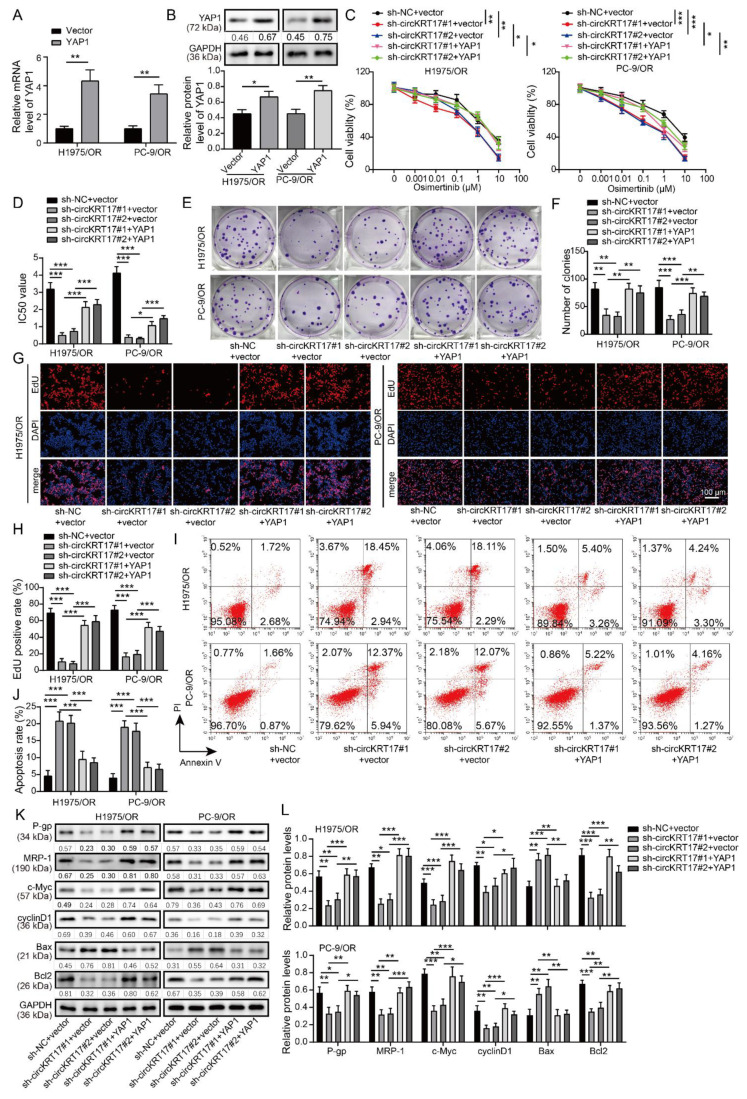
YAP1 overexpression reversed the promotive effects of circKRT17 knockdown on the sensitivity of osimertinib-resistant LUAD cells to osimertinib. (**A**,**B**) Relative mRNA and protein expression of YAP1 in treated H1975/OR and PC-9/OR cells (**C**,**D**) Osimertinib IC50 value of H1975/OR and PC-9/OR cells transfected with shcircKRT17#1 or shcircKRT17#2 plus YAP1 overexpression plasmid. (**E**,**F**) Colony formation assay and (**G**,**H**) EdU immunofluorescence staining were performed to detect cell proliferation after treatment with shcircKRT17#1 or shcircKRT17#2 plus YAP1 overexpression plasmid. (**I**,**J**) After co-transfection with shcircKRT17#1 or shcircKRT17#2 plus YAP1 overexpression plasmid, H1975/OR and PC-9/OR cells were subjected to apoptosis analysis using flow cytometry. (**K**,**L**) Western blot analysis of P-gp, MRP-1, c-Myc, CyclinD1 Bax, and Bcl2 in H1975/OR and PC-9/OR cells transfected with shcircKRT17#1 or shcircKRT17#2 plus YAP1 overexpression plasmid. * *p* < 0.05, ** *p* < 0.01 and *** *p* < 0.001. File S1: Original western blots.

Next, rescue assays were designed to uncover the function of the circKRT17/YAP1 axis in the resistance of H1975/OR and PC-9/OR cells to osimertinib. A YAP1 overexpression plasmid was used to overexpress YAP1, qRT-PCR and western blotting results indicated a remarkable elevation of YAP1 mRNA and protein in both H1975/OR and PC-9/OR cells transfected with YAP1 overexpression plasmid compared to vector group (Figure 7A,B). Knockdown of circKRT17 significantly decreased the osimertinib IC_50_ value of H1975/OR and PC-9/OR cells, while YAP1 overexpression could restore the osimertinib IC_50_ value (Figure 7C,D). By using the colony formation assay (Figure 7E,F) and EdU immunofluorescence staining (Figure 7G,H), we revealed the dramatic repression of cell proliferation in circKRT17-silenced cells, while YAP1 overexpression abrogated the inhibitive impacts of circKRT17 silencing on cell proliferation. The promotive effects of circKRT17 knockdown on cell apoptosis were also abrogated by YAP1 overexpression (Figure 7I,J). Knockdown of circKRT17 decreased the levels of multidrug resistance-related proteins (P-gp and MRP-1) and pro-survival proteins (c-Myc, Cyclin D1, and Bcl2) and increased the expression of pro-apoptotic protein Bax; moreover, the alterations of these proteins induced by circKRT17 knockdown were reversed by YAP1 overexpression (Figure 7K,L). These findings suggested that circKRT17 regulated the sensitivity of LUAD cells to osimertinib by YAP1 in vitro.

### 3.8. Knockdown of CIRCKRT17 Enhanced the Suppressive Effects of Osimertinib on Tumor Growth In Vivo by Inhibiting YAP1 Signaling

**Figure 8:**
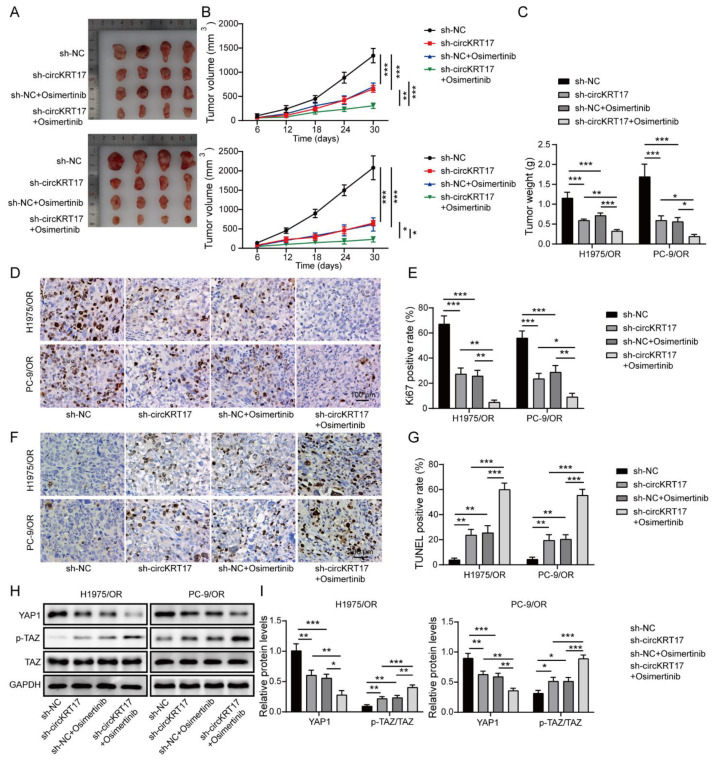
Knockdown of circKRT17 enhanced the suppressive effects of osimertinib on tumor growth in vivo by inhibiting YAP1. H1975/OR and PC-9/OR cells stably transfected with shcircKRT17 were delivered into mice with or without the treatment of osimertinib. (**A**) Representative images of xenograft tumors from shNC, shcircKRT17, shNC + osimertinib, AND shcircKRT17 + osimertinib groups. (**B**,**C**) Tumor volume and weight from different groups. (**D**,**E**) Ki-67 staining and (**F**,**G**) TUNEL staining were carried out to assess tumor cell proliferation and apoptosis in xenograft tumor sections from different groups. (**H**,**I**) Protein level analysis of YAP1, p-TAZ, and TAZ in xenograft tumors from different groups. * *p* < 0.05, ** *p* < 0.01 and *** *p* < 0.001. File S1: Original western blots.

H1975/OR and PC-9/OR cells stably transfected with shNC or shcircKRT17 were injected into nude mice. When the tumor volume reached 50 mm^3^, mice were treated with osimertinib (25 mg/kg/d) or noting by oral gavage for 14 days. Knockdown of circKRT17 alone significantly decreased the volume and weight of tumors, combined with osimertinib treatment could further decrease the volume and weight of tumors (Figure 8A–C). By using Ki-67 staining, we proved that the knockdown of circKRT17 alone significantly repressed tumor cell proliferation, and the combination of osimertinib and shcircKRT17 further repressed tumor cell proliferation (Figure 8D,E). We also showed that the knockdown of circKRT17 alone significantly promoted tumor cell apoptosis (as demonstrated by TUNEL staining), and the combination of osimertinib and shcircKRT17 further promoted tumor cell apoptosis (Figure 8F,G). Moreover, knockdown of circKRT17 alone induced the downregulation of YAP1 and the upregulation of p-TAZ was enhanced by the combined treatment with osimertinib (Figure 8H,I). These findings suggested that the knockdown of circKRT17 improved the therapeutic effects of osimertinib on LUAD in vivo by inhibiting YAP1.

## 4. Discussion

Osimertinib has improved the prognosis of LUAD patients to a certain degree in the last few years; nevertheless, its therapeutic outcomes are largely limited by the acquired resistance [25]. Hence, it is critical to develop novel selective targets that can enhance the sensitivity of LUAD cells to osimertinib. Herein, we assessed the specificity and efficacy of METTL3/circKRT17/EIF4A3/YAP1 signaling axis in regulating the sensitivity of LUAD cells to osimertinib in both in vitro and in vivo. Based on our results, we believe this axis is a novel target for increasing the sensitivity of LUAD cells to osimertinib.

KRT17 was revealed to be highly presented in cancer tissues and has been adopted as a clinical biomarker for various human tumors [26,27,28]. It was also proven to facilitate lung cancer cell proliferation and invasion, and elevated KRT17 is closely related to a poor prognosis [29,30]. In a recent circRNAs microarray analysis performed by Chen et al., circKRT17 was demonstrated to be elevated in osimertinib-resistant lung cancer cell lines [16], implying circKRT17 might play a role in the osimertinib resistance of LUAD. However, no study has verified the functional role of circKRT17 in the osimertinib resistance of LUAD. Accumulating evidence has shown the important role of circRNAs in regulating the biological events of LUAD, such as proliferation, metastasis, and glycolysis [31,32,33]. However, only one paper focused on the role of circRNAs in the osimertinib resistance of LUAD, suggesting that circ_0005576 promotes osimertinib resistance of LUAD by miR-512-5P/IGF1R axis [34]. Herein, we confirmed the elevation of circKRT17 in osimertinib-resistant LUAD, and circKRT17 knockdown could increase the sensitivity of LUAD cells to osimertinib. This study is the first one to report the function of circKRT17 in tumorigenesis and drug resistance of LUAD.

As one of the most abundant RNA modifications, m6A dysregulation has been identified by current studies to contribute to multiple human diseases, especially for certain cancers [35,36]. Moreover, m6A modification has been found to facilitate tumor progression in most of the cases [37,38], but recent studies have also shown its role in suppressing tumor progression [39]. METTL3, as an m6A “writer”, affects almost all kinds of biological behaviors of various tumors by initiating m6A methylation on target mRNA [40,41,42]. Recently, elevated METTL3 has been found in the TCGA-LUAD database, which may be a new biomarker for the prognosis of LUAD patients [20]. Moreover, METTL3 could facilitate the progression of NSCLC by mediating the m6A modification of Bcl2 mRNA [43]. In addition, METTL3 has been proven to affect the resistance of human cancers to multiple drugs, including adriamycin, sorafenib, and gefitinib [44,45,46]. Nevertheless, no study has studied MTTLE’s role in regulating the resistance to LUAD. Previous studies focused on m6A-modified mRNA, but in fact, m6A-modified circRNAs also play a key role in tumorigenesis [47,48]. Herein, we reported an elevated METTL3 in LUAD, in line with previous studies [20,43]. Moreover, we found that METTL3 could increase the stability of circKRT17 by initiating m6A modification on it and, thus, affecting the sensitivity of LUAD cells to osimertinib.

Yes-associated protein 1 (YAP1) has been proven to function as a key modulator in tumor cell proliferation, metastasis, and stem cell activity since it was first identified in 1994 [49,50,51]. YAP1 was revealed to be elevated in LUAD, and YAP1 inhibition could repress the impacts of the inflammation on LUAD cell proliferation, invasion, and migration [52]; nevertheless, the precise mechanisms of YAP1 in LUAD remain unclear. Moreover, METTL3 was found to facilitate YAP translation by modulating miR-1914-3p to initiate NSCLC cisplatin resistance and metastasis [21]. YAP1 was also proven to be related to the multidrug resistance of lung cancer by CD74-related signaling pathways [53]. Additionally, m6A-induced circ1662 was proven to promote colorectal cancer cell metastasis by promoting YAP1 nuclear localization [54]. Our study indicated that circKRT17 might promote YAP1 nuclear localization and, thus, regulate the osimertinib resistance of LUAD.

## 5. Conclusions

In summary, our results illustrated that circKRT17 silencing elevated the sensitivity of LUAD to osimertinib, indicating a key role of circKRT17 in modulating LUAD osimertinib resistance. Mechanistically, METTL3 enhanced the stability of circKRT17 by initiating m6A modification and, thus, promoting YAP1 nuclear localization through the recruitment of EIF4A3. These collective data provide novel insights into therapeutic strategies for osimertinib-resistant LUAD patients. CircKRT17 may act as a biomarker of osimertinib resistance of LUAD and a promising target for osimertinib-resistant LUAD treatment.

## Data Availability

All data generated or analyzed during this study are included in this article. The datasets used and/or analyzed during the current study are available from the corresponding author upon reasonable request.

## Supplementary Materials

The following supporting information can be downloaded at: https://www.mdpi.com/article/10.3390/cancers14225582/s1, File S1: Uncropped western blots for figures.
