# Supplementary figures and images for: N6-Methyladenosine Modification of CIRCKRT17 Initiated by METTL3 Promotes Osimertinib Resistance of Lung Adenocarcinoma by EIF4A3 to Enhance YAP1 Stability
Source: Cancers (Basel). 2022 Nov 14;14(22):5582. doi: 10.3390/cancers14225582 (PMC9688051; doi:10.3390/cancers14225582)

Fig. 2J

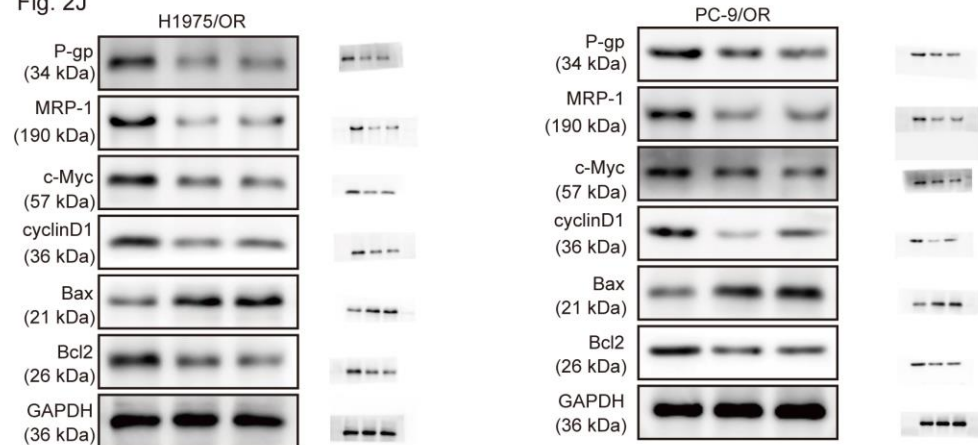

Fig. 3D

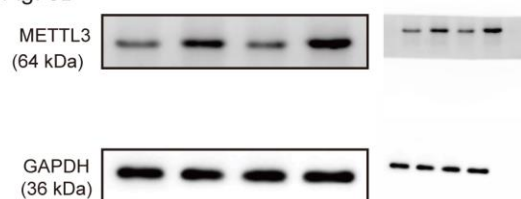

Fig. 3G

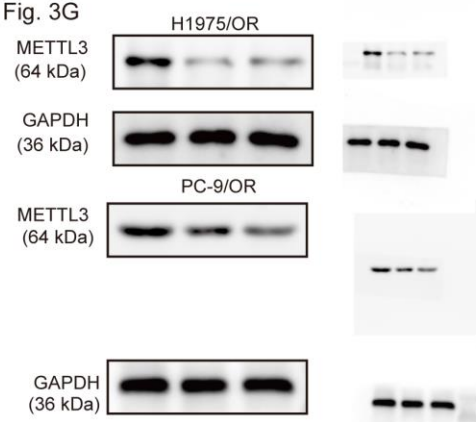

Fig. 5D

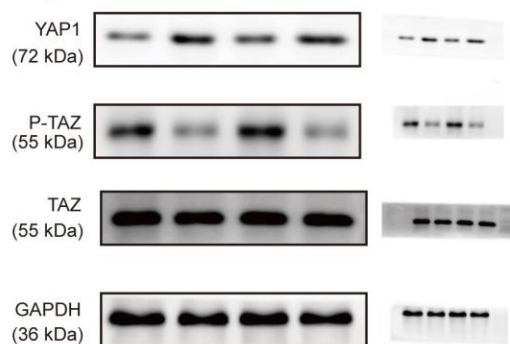

Fig. 6A

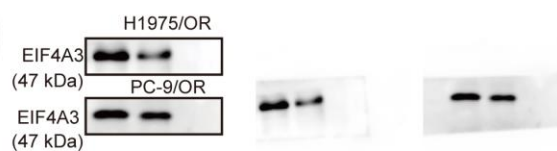

Fig. 4J

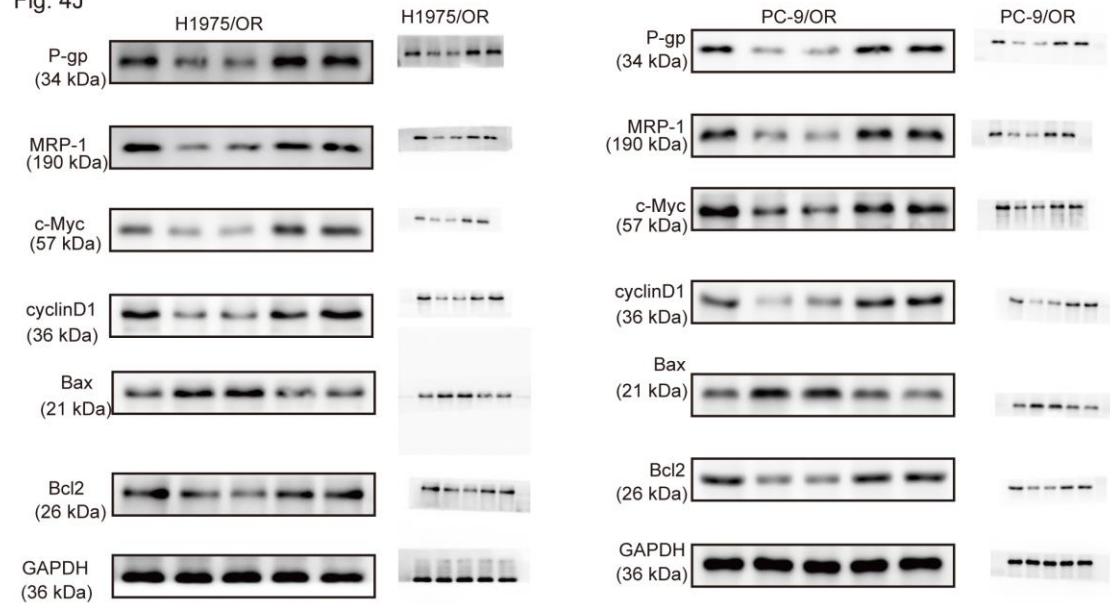

Fig. 5G

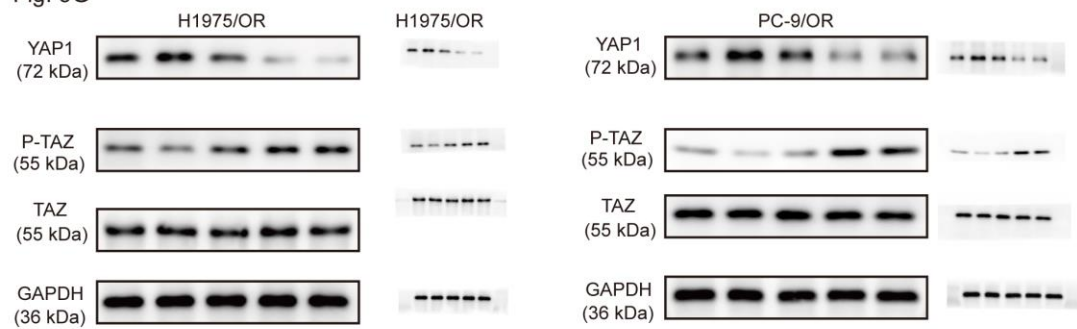

Fig. 7B

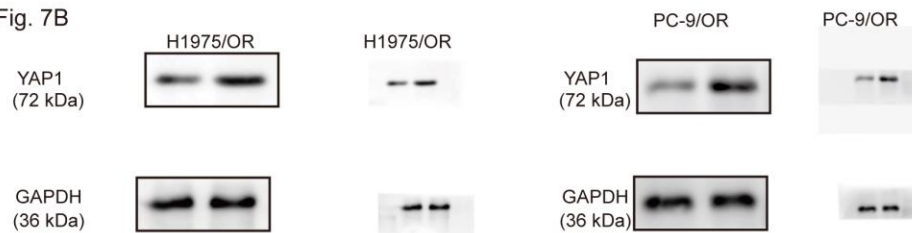

Fig. 6A

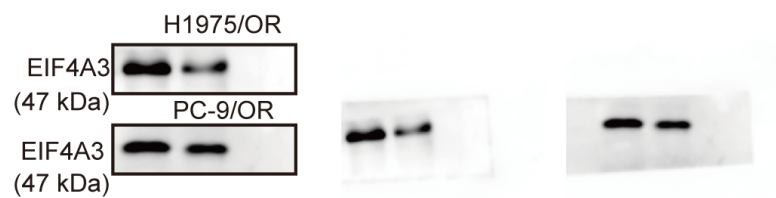

Fig. 7K

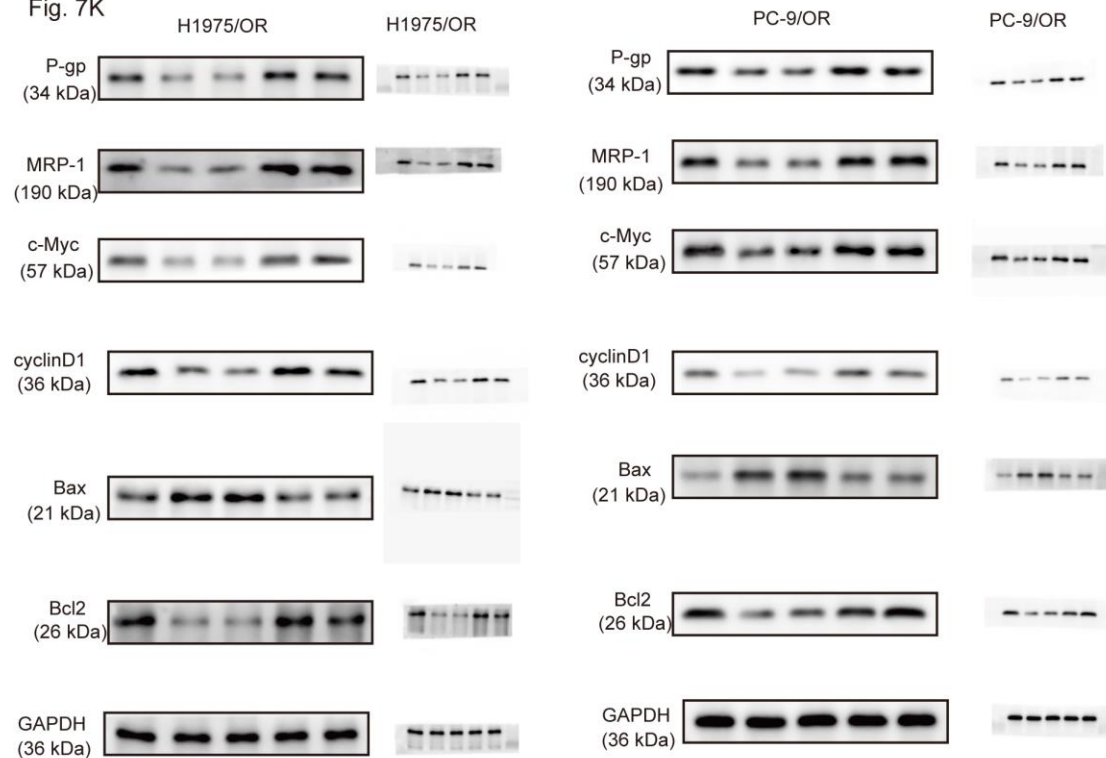

Fig. 8H

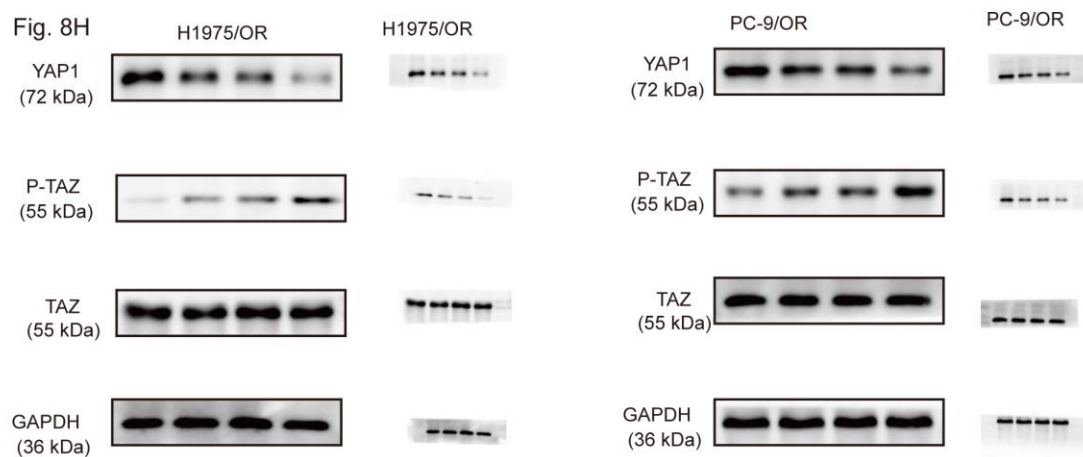

Supplement: Supplementary file 1 [file cancers-14-05582-s001.zip › cancers-1927233-SI.pdf]
